# Coenzyme Q_10_ prevents hepatic fibrosis, inflammation, and oxidative stress in a male rat model of poor maternal nutrition and accelerated postnatal growth^1^

**DOI:** 10.3945/ajcn.115.119834

**Published:** 2015-12-30

**Authors:** Jane L Tarry-Adkins, Denise S Fernandez-Twinn, Iain P Hargreaves, Viruna Neergheen, Catherine E Aiken, Malgorzata S Martin-Gronert, Josie M McConnell, Susan E Ozanne

**Affiliations:** 2 University of Cambridge Metabolic Research Laboratories and Medical Research Council Metabolic Diseases Unit, Institute of Metabolic Science, Addenbrooke’s Treatment Centre, Addenbrooke’s Hospital, Cambridge, United Kingdom; and; 3 Neurometabolic Unit, National Hospital, University College London, London, United Kingdom

**Keywords:** developmental programming, liver disease, coenzyme Q, low birth weight, accelerated postnatal growth

## Abstract

**Background:** It is well established that low birth weight and accelerated postnatal growth increase the risk of liver dysfunction in later life. However, molecular mechanisms underlying such developmental programming are not well characterized, and potential intervention strategies are poorly defined.

**Objectives**: We tested the hypotheses that poor maternal nutrition and accelerated postnatal growth would lead to increased hepatic fibrosis (a pathological marker of liver dysfunction) and that postnatal supplementation with the antioxidant coenzyme Q_10_ (CoQ_10_) would prevent this programmed phenotype.

**Design:** A rat model of maternal protein restriction was used to generate low-birth-weight offspring that underwent accelerated postnatal growth (termed “recuperated”). These were compared with control rats. Offspring were weaned onto standard feed pellets with or without dietary CoQ_10_ (1 mg/kg body weight per day) supplementation. At 12 mo, hepatic fibrosis, indexes of inflammation, oxidative stress, and insulin signaling were measured by histology, Western blot, ELISA, and reverse transcriptase–polymerase chain reaction.

**Results:** Hepatic collagen deposition (diameter of deposit) was greater in recuperated offspring (mean ± SEM: 12 ± 2 μm) than in controls (5 ± 0.5 μm) (*P* < 0.001). This was associated with greater inflammation (interleukin 6: 38% ± 24% increase; *P* < 0.05; tumor necrosis factor α: 64% ± 24% increase; *P* < 0.05), lipid peroxidation (4-hydroxynonenal, measured by ELISA: 0.30 ± 0.02 compared with 0.19 ± 0.05 μg/mL per μg protein; *P* < 0.05), and hyperinsulinemia (*P* < 0.05). CoQ_10_ supplementation increased (*P* < 0.01) hepatic CoQ_10_ concentrations and ameliorated liver fibrosis (*P* < 0.001), inflammation (*P* < 0.001), some measures of oxidative stress (*P* < 0.001), and hyperinsulinemia (*P* < 0.01).

**Conclusions:** Suboptimal in utero nutrition combined with accelerated postnatal catch-up growth caused more hepatic fibrosis in adulthood, which was associated with higher indexes of oxidative stress and inflammation and hyperinsulinemia. CoQ_10_ supplementation prevented liver fibrosis accompanied by downregulation of oxidative stress, inflammation, and hyperinsulinemia.

## INTRODUCTION

In 1992, Hales and Barker (1) proposed the “thrifty phenotype hypothesis,” which postulated that in response to suboptimal in utero nutrition, the fetus alters its organ structure and adapts its metabolism to ensure immediate survival. This occurs through the sparing of vital organs (e.g., the brain) at the expense of others, such as the liver, thus increasing the risk of metabolic disease such as liver dysfunction in later life (2–4). This risk is exacerbated if a suboptimal uterine environment is followed by rapid postnatal growth (5, 6).

Nonalcoholic fatty liver disease (NAFLD)^4^ is the hepatic manifestation of the metabolic syndrome. Aspects of the metabolic syndrome, including NAFLD, have been linked to exposure to suboptimal early-life environments (7, 8). Although the incidence of NAFLD is high (9), the associated morbidity is low if there is no progression to hepatic fibrosis. Progression to fibrosis is indicative of the clinically important subtype of patients with NAFLD who have a high chance (20%) of developing frank liver cirrhosis and subsequent liver failure (10). At present, it is unknown why this progression occurs only in a subset of individuals. The development of an intervention that prevents these changes from accumulating could improve the prognosis of patients who develop NAFLD later in life.

Increased oxidative stress is a common consequence of developmental programming (11). Increased reactive oxygen species (ROS) have been strongly implicated in the etiology of hepatic fibrosis. Several animal studies have focused on antioxidant therapies to prevent the deleterious phenotypes of developmental programming (12–14); however, the doses used are not recommended for use in humans. In practice, a suboptimal intrauterine environment is often recognized retrospectively (i.e., after delivery). Thus, it is important to address potential beneficial effects of targeted postnatal interventions.

Coenzyme Q (CoQ_10_) is a benzoquinone ring linked to an isoprenoid side-chain. The isoform containing 9 isoprenoid units (CoQ_9_) is most abundant in rodents, whereas CoQ_10_ (10 isoprenoid units) is the most common in humans. When oxidized, CoQ_10_ shuttles electrons between mitochondrial complexes I and III and complexes II and III. Reduced CoQ_10_ is the most abundant endogenous cellular antioxidant (15) and is a safe and effective therapeutic antioxidant (16, 17). We have also shown that postnatal CoQ_10_ supplementation prevents developmentally programmed accelerated aging in rat aortas (18) and hearts (19).

Liver is one of a few tissues to take up dietary CoQ_10_ (20), and CoQ_10_ supplementation has been previously investigated as a potential therapy to prevent the progression of NAFLD in humans (21). In this study, we aimed to *1*) investigate the effects of poor maternal nutrition and rapid postnatal catch-up growth on hepatic CoQ_9_ concentrations and molecular pathways leading to proinflammatory changes and development of fibrosis and *2*) determine whether a clinically relevant dose of dietary CoQ_10_ could correct any observed hepatic fibrosis.

## METHODS

### Animal experimentation

All procedures involving animals were conducted under the British Animals (Scientific Procedures) Act (1986) and underwent ethical review by the University of Cambridge Animal Welfare and Ethical Review Board. Stock animals were purchased from Charles River, and dams were produced from in-house breeding from stock animals. 

Pregnant Wistar rats were maintained at room temperature in specific-pathogen-free housing with the use of individually ventilated cages with environmental enrichment. The dams were maintained on a 20% protein diet (control) or an isocaloric low-protein (8%) diet, as previously described (22). Access to diets and water was provided ad libitum. All rats used in this study were specific-pathogen-free housed individually at 22°C on a controlled 12:12-h light-dark cycle. Diets were purchased from Arie Blok. 

The day of birth was recorded as day 1 of postnatal life. Pups born to low-protein-diet–fed dams were cross-fostered to control-fed mothers on postnatal day 3 to create a recuperated litter. Each recuperated litter was standardized to 4 male pups at random to maximize their plane of nutrition. The control group consisted of the offspring of dams fed the 20%-protein diet and suckled by 20% protein–fed dams. Each control litter was culled to 8 pups as a standard. To minimize stress to the pups when cross-fostered, they were transferred with some of their own bedding. Body weights were recorded at postnatal days 3 and 21 and at 12 mo. At 21 d, 2 males per litter were weaned onto standard laboratory feed pellets (Special Diet Services) and the other 2 were weaned onto the same diet supplemented with CoQ_10_ to give a dose of 1 mg/kg body weight per day. Diets were given in the home cage. Rat pups were fed these diets until 12 mo of age, at which time all rats were killed by carbon dioxide asphyxiation at ∼1000. Post mortem, liver tissue was removed, weighed, and snap-frozen in liquid nitrogen and then stored at −80°C until analysis. A further portion of liver tissue (the same area for each sample) was removed and fixed in formalin for histologic assessment. 

For all measurements, 1 pup per litter was used, thus “*n*” represents number of litters. Ten litters per group were used in this study based on power calculation with the use of previous data from our studies of RNA expression in postnatal tissues from programmed animals. Rat numbers were calculated to give 80% power to detect a 20% difference between groups at the *P* < 0.05 level. Only male rats were used in this study.

### CoQ_10_ diet preparation

A dose of 1 mg CoQ_10_/kg body weight per day was used in this study, which was administered via the diet (18). This was achieved by appropriate CoQ_10_ supplementation of laboratory feed pellets, as we described previously (18, 19). Diet was prepared twice a week throughout the study.

### CoQ_10_, lipid profile, glucose, and insulin analysis

Total liver ubiquinone (CoQ_9_ and CoQ_10_) was quantified by reverse-phase HPLC with UV detection at 275 nm, as described previously (18, 19). Serum was obtained as detailed previously (18), and blood from the tail vein collected into EDTA tubes and centrifuged for 3 min at 3000 rpm at 4° Celsius to isolate plasma. Fasted blood glucose measurements were obtained by using a glucose analyzer (Hemocue). The serum lipid profile and fasted plasma insulin analyses were performed by using an auto-analyzer (the Wellcome Trust–supported Cambridge Mouse Laboratory). Liver triglyceride concentrations were determined by using the Folch assay (23). Briefly, liver samples were homogenized in a 2:1 ratio of chloroform:methanol. The distinct lipid phase was removed after centrifugation, and lipid weight was quantified after the solvent was removed by evaporation.

### Histologic assessment

Liver samples were fixed in formalin, paraffin-embedded, and sectioned to a 6-μm thickness by using a microtome. Picro Sirius Red staining was used to stain for fibrosis. Cell-D software (Olympus Soft Imaging Solutions) was used to quantify the thickness of fibrosis around all visible hepatic vessels (including all arteries and veins) from 1 section per sample. This sample was taken at the same point (20 sections for each sample) by using a nonbiased grid sampling method. All analyses were performed at 10× magnification by using an Olympus microscope (Olympus Soft Imaging Solutions). All analyses were performed blinded.

### Mitochondrial electron transport chain complex activities

Activities of complex I [NAD(H): ubiquinone reductase; Enzyme Commission (EC) 1.6.5.3], complexes II–III (succinate: cytochrome *c* reductase; EC 1.3.5.1 + EC 1.10.2.2), and complex IV (cytochrome oxidase; EC 1.9.3.1) as well as citrate synthase (EC 1.1.1.27) were assayed as described previously (18, 19).

### Protein analysis

Protein was extracted and assayed as described previously (18). Protein (20 μg) was loaded onto 10%, 12%, or 15% polyacrylamide gels, dependent on the molecular weight of the protein to be measured. The samples were electrophoresed and transferred to polyvinylidene fluoride membranes (18) and detected with the use of the following dilutions of primary antibody: insulin receptor substrate 1 (IRS-1; 1:1000; Millipore); phosphoinositide-3-kinase, p110-β (p110-β); insulin receptor β (IR-β); protein kinase-ζ (1:200; Santa-Cruz); Akt-1 and Akt-2 (1:1000; New England Biolabs); phosphoinositide-3-kinase, p85-α (p85-α 1:5000; Upstate); cytochrome P450-2E1 (CYP2E1) and Il-6 (1:1000; Abcam); Tnf-α (1:1000; Cell Signaling Technology); and anti-rabbit IgG secondary antibodies (1:20,000; Jackson Immunoresearch Laboratories). Equal protein loading was confirmed by staining electrophoresed gels with Coomassie Blue (Bio-Rad) to visualize total protein.

### Gene expression

RNA was extracted by using a miReasy mini kit (Qiagen) following the manufacturer’s instructions, as detailed previously (19). A DNase digestion step was performed to ensure no genetic DNA contamination. RNA (1 μg) was used to synthesize complementary DNA by using oligo-dT-adaptor primers and Moloney murine leukemia virus reverse transcriptase (Promega). Gene expression was determined by using custom-designed primers (Sigma) and SYBR Green reagents (Applied Biosystems). Primer sequences are presented in **Table 1**. Quantification of gene expression was performed with the use of a Step One Plus reverse transcriptase–polymerase chain reaction machine (Applied Biosystems). Equal efficiency of the reverse transcription of RNA from all groups was confirmed through quantification of expression of the housekeeping gene β-actin (*Actb*). The expression of *Actb* did not differ between groups (effect of maternal diet, *P* = 0.9; effect of CoQ_10_ supplementation, *P* = 0.8; control: 153 ± 32; recuperated: 144 ± 24; control CoQ_10_: 143 ± 12; and recuperated CoQ_10_: 157 ± 21 average copy numbers).

**TABLE 1:** Primers^1^

	Sequence		
Primer	F	R	Product size, bp
*Col1a1*	GCCTCCCAGAACATCACCTA	GCAGGGACTTCTTGAGGTTG	82
*Tgfb*	TGCCCTCTACAACCAACACA	CTTGCGACCCACGTAGTAGA	100
*Mcp1*	TGGACCAGAACCAAGTGAGA	TGCTGAAGTCCTTAGGGTTGA	71
*Acta2*	GACATCAGGAAGGATCTCTATGC	TCTCCTTCTGCATCCTGTCA	88
*Des*	GGAGGAGATCCGACACCTAA	ACATCCAAGGCCATCTTCAC	87
*Gfap*	GAGTTACCAGGAGGCACTCG	GGGCCATCTCCTCCTTGAG	65
*Clec4f*	ACGGAGAGCGTGAAGACTGT	CTTGCACACCCAGTTGTAGG	83
*Cd68*	AAAGCTTCTGTTGCGGAAAT	GAGCAGGTCCAGGTGAATTG	62
*Mmp2*	GGAAAGAGGATACCCCAAGC	TCCAGTTAAAGGCAGCGTCT	80
*Mmp9*	TTGGGTCTAGGCTCAGAGGT	AGATACGTTCCCGGCTGAT	88
*Timp1*	CTGAGAAGGGCTACCAGAGC	TATTGCCAGGTGCACAAATC	70
*Timp2*	GGATGGACTGGGTCACAGAG	GCGCAAGAACCATCACTTCT	85
*Gp91^phox^*	CGAAGCCTTGGCTAAAACTCT	TCCTTGTTGAAGATGAAGTGGA	87
*P22^phox^*	GTGAGCAGTGGACTCCCATT	GTAGGTGGCTGCTTGATGGT	76
*P47^phox^*	TGTGACACCCTCTCACAGACA	GTCGCATTTTCCCTCCTTTA	96
*P67^phox^*	CCGATAACCGGACAACAGAG	CAGGTCTTCTGGCTGGGTAG	72
*P40^phox^*	GATGTGGGACTCATGGTGAA	AATTGTCCTTCTGGGTGACG	91
*Nrf2*	AGCAAGACTTGGGCCACTTA	GATGGAGGTTTCTGTCGTTTTC	78
*Hmox1*	TAACCAGGATCTCCCCAAGA	TTAGAGTGCTGTGGCAGGTG	73
*Gpx1*	CACCCGCTCTTTACCTTCCT	CGGGGACCAAATGATGTACT	75
*Gst*	TCTTGTTGGCAACCAACTCA	AGTCAGACAGCACAGGAGCA	92
*Nqo1*	TGGAGACTGTCTGGGAGGAG	TCCTGCCTGGAAGTTTAGGT	74
*Actb*	ATGCTGCGTCTGGACTG	CTCCAGTGTGGTGAA	85

1 *Acta2*, α-smooth muscle actin 2; *Actb*, β-actin; *Cd68*, cluster of differentiation 68; *Clec4f*, C-type lectin-domain family 4; *Col1a1*, collagen type 1, α1; *Des*, desmin; F, forward; *Gfap*, glial fibrillary acidic protein; *Gpx1*, glutathione peroxidase 1; *Gst*, glutathione synthetase; *Hmox1*, heme oxygenase 1; *Mcp1*, monocyte chemoattractant protein 1; *Mmp*, matrix metalloproteinase; *Nqo1*, NAD(P)H dehydrogenase, quinone 1; *Nrf2*, nuclear factor, erythroid 2–like 2; R, reverse; *Tgfb*, transforming growth factor β *Timp*, tissue inhibitor of matrix metalloproteinases.

### Mitochondrial DNA copy number

Total DNA was extracted using a phenol/chloroform extraction protocol (24). Mitochondrial DNA copy number analysis was performed as described previously (25).

### 4-Hydroxynonenal and 3-nitrotyrosine analysis

Protein nitrotyrosination was assayed by using a Nitrotyrosine ELISA kit (MitoSciences), according to the manufacturer’s instructions. Lipid peroxidation was analyzed by using an OxiSelect HNE Adduct ELISA kit (Cambridge Biosciences), according to the manufacturer’s instructions.

### Statistical analysis

Data were analyzed by using a 2-factor ANOVA with maternal diet and CoQ_10_ supplementation as the independent variables. Post hoc testing was carried out when appropriate and is indicated in the text accordingly. Data are represented as means ± SEMs. All statistical analyses were performed with the use of Statistica 7 software (Statsoft), and for all tests, *P* values <0.05 were considered significant. Data were checked for normal distribution. In all cases, “*n*” refers to the number of litters (with 1 rat used from each litter).

## RESULTS

### Anthropometric measurements

Recuperated offspring were born smaller than control rats (6.3 ± 0.2 compared with 7.4 ± 0.2 g; *P* < 0.001) and underwent rapid postnatal catch-up growth so that their weights were similar to those of the control offspring at postnatal day 21 (52.2 ± 0.9 compared with 50.7 ± 1.2 g). At 12 mo of age, there was no effect of maternal diet or CoQ_10_ supplementation on body weights or absolute liver weights (**Table 2**).

**TABLE 2:** Effect of in utero protein restriction, accelerated postnatal growth, and CoQ_10_ supplementation on rat pup body and liver weights^1^

	Control	Recuperated	Control CoQ_10_	Recuperated CoQ_10_
Body weight at 12 mo, g	956.3 ± 25.2	920.3 ± 28.8	979.6 ± 35.1	935.7 ± 29.9
Absolute liver weight at 12 mo, g	27.5 ± 1.4	28.4 ± 1.2	28.1 ± 1.2	29.5 ± 1.3

1 Values are means ± SEMs; *n* = 10/group. Data were analyzed by using 2-factor ANOVA and Duncan’s post hoc testing, where appropriate. No significant differences between groups were reported. CoQ_10_, coenzyme Q.

### Dietary CoQ_10_ supplementation leads to greater hepatic CoQ_9_ and CoQ_10_ concentrations

Recuperated hepatic CoQ_9_ and CoQ_10_ concentrations were unaltered compared with those in control rats (**Table 3**). However, CoQ_9_ and CoQ_10_ concentrations were greater (*P* < 0.01) when supplemented with CoQ_10_ (Table 3).

**TABLE 3:** Effect of in utero protein restriction, accelerated postnatal growth, and CoQ_10_ supplementation on serum and plasma measurements^1^

	Control	Recuperated	Control CoQ_10_	Recuperated CoQ_10_	Effect of CoQ_10_
Hepatic CoQ_9_, pmol/mg protein	494 ± 28	497 ± 24	872.4 ± 79**	1071 ± 266**	0.003
Hepatic CoQ_10_, pmol/mg protein	80 ± 7	78 ± 6	146 ± 17**	102 ± 11**	0.002
Fasting plasma glucose, mmol/L	5.6 ± 0.2	5.3 ± 0.1	5.4 ± 0.3	5.24 ± 0.2	0.9
Serum cholesterol, mmol/L	4.8 ± 0.5	4.9 ± 0.4	6.1 ± 0.7*	5.9 ± 0.6*	0.04
Serum trigyclerides, mmol/L	3.8 ± 0.05	3.8 ± 0.4	4.2 ± 0.6*	5.7 ± 0.7*	0.02
Hepatic triglyceride content, g	2.0 ± 0.2	2.3 ± 0.2	2.0 ± 0.1	2.0 ± 0.2	0.3

1 Values are means ± SEMs; *n* = 10/group. Data were analyzed by using 2-factor ANOVA and Duncan’s post hoc testing, where appropriate. The overall effects of maternal diet and interaction between maternal diet and CoQ_10_ supplementation were not significant for any of the variables reported in the table. *^,^**Effect of CoQ_10_: *P < 0.05, **P < 0.01. CoQ_10_, coenzyme Q.

### CoQ_10_ supplementation ameliorates hepatic fibrosis and inflammation induced by poor maternal nutrition

**FIGURE 1:**
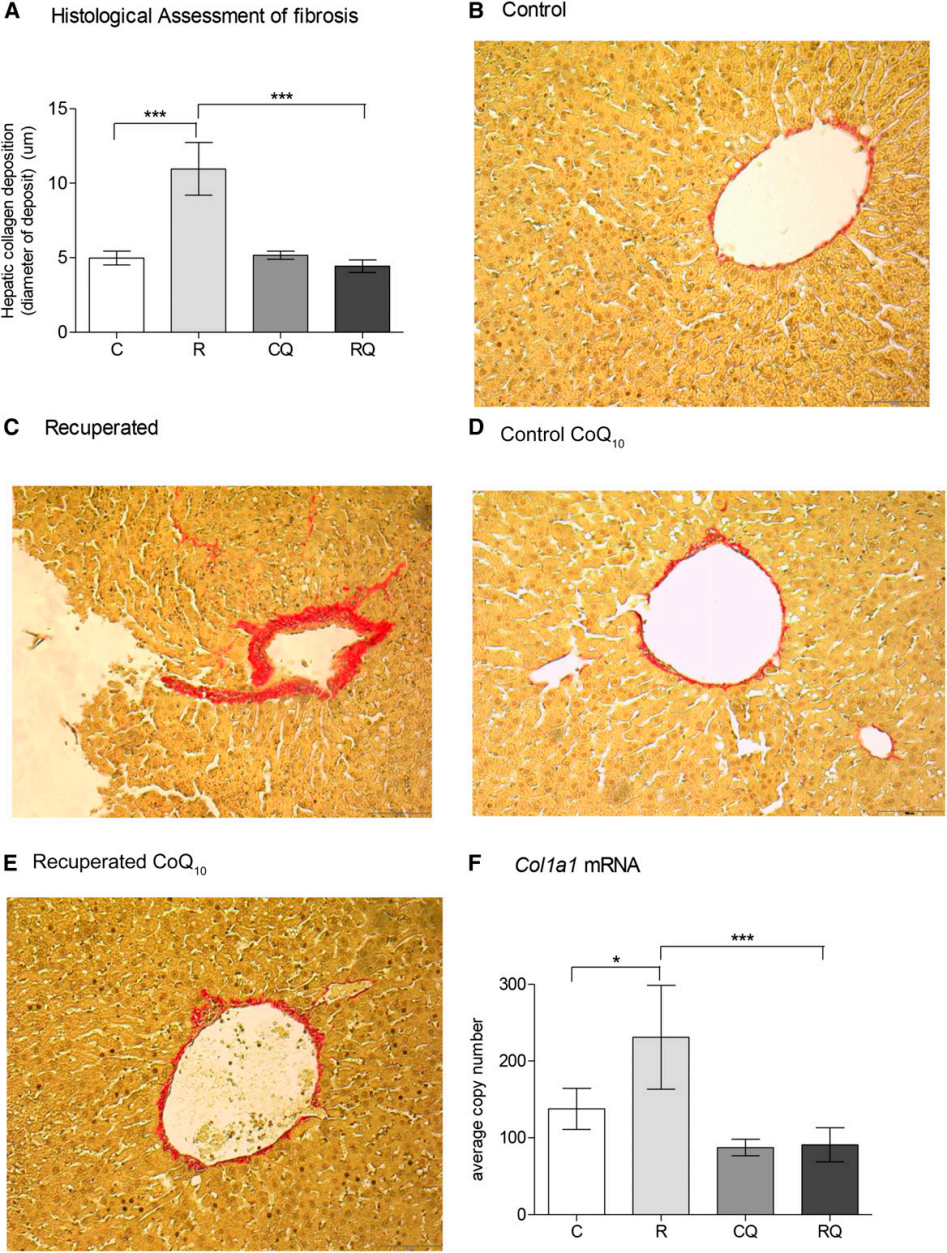
Effect of in utero protein restriction, accelerated postnatal growth, and CoQ_10_ supplementation on hepatic fibrosis, quantified by measurement of collagen (A), in 12-mo-old male rat livers (B–E). (F) mRNA expression of *Col1a1*. Values are as means ± SEMs; *n* = 10/group; 10/10 rats used. *^,^***C compared with R and R compared with RQ: *P < 0.05, ***P < 0.001. Interaction between in utero protein restriction, accelerated postnatal growth, and CoQ_10_ supplementation (statistical interaction): *P* = 0.001 (A and F). Data were analyzed by using 2-factor ANOVA and Duncan’s post hoc testing, where appropriate. C, control; *Col1a1* = collagen type 1, α1; CoQ_10_, coenzyme Q; CQ, control CoQ_10_; R, recuperated; RQ, recuperated CoQ_10_.

Recuperated offspring showed greater (*P* < 0.001) collagen deposition (**Figure 1**A) than did control offspring (Figure 1B, C). CoQ_10_ supplementation prevented this effect of maternal diet (*P* < 0.001; interaction between maternal diet and CoQ_10_ supplementation, *P* = 0.001) (Figure 1A, D, E). Collagen type 1, α1 (*Col1a1*), mRNA expression was also greater (*P* < 0.05) in recuperated offspring (Figure 1F) and was reduced (*P* < 0.05) by CoQ_10_ supplementation (interaction between maternal diet and CoQ_10_ supplementation, *P* = 0.001) (Figure 1F). 

**FIGURE 2:**
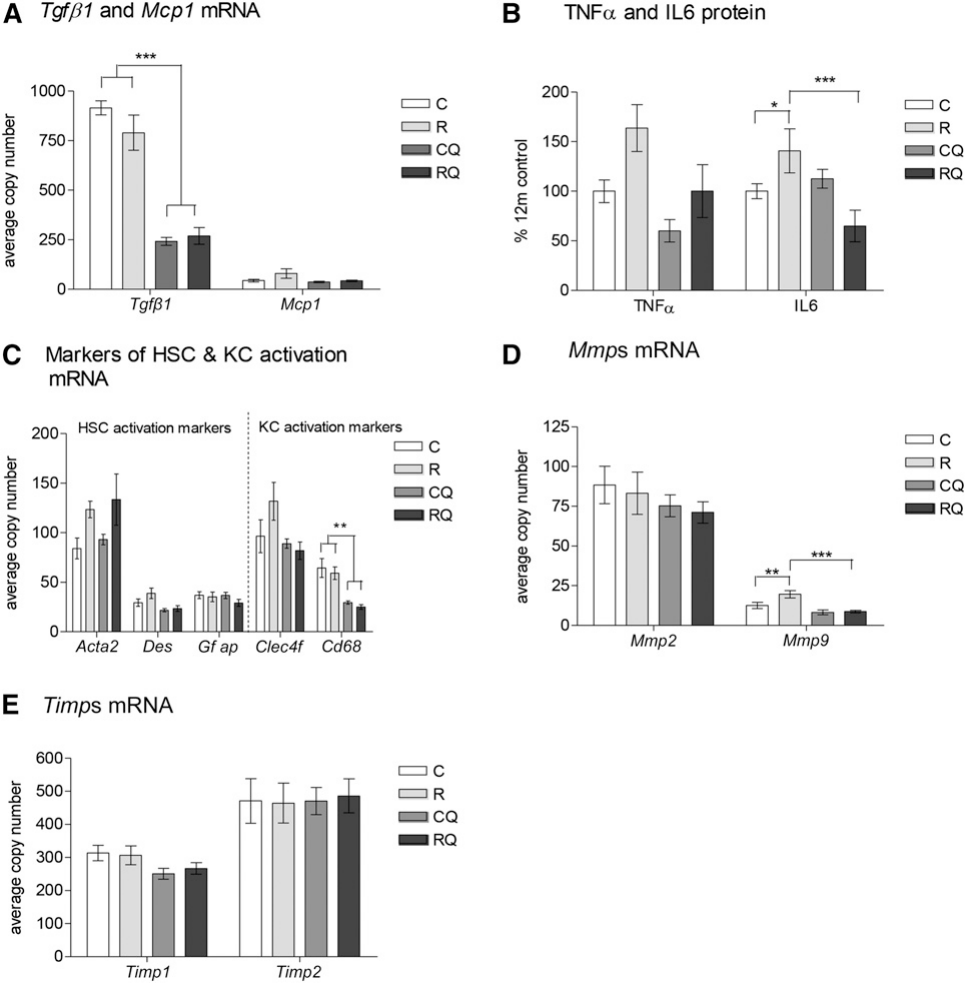
Effect of in utero protein restriction, accelerated postnatal growth, and CoQ_10_ supplementation on inflammatory markers *Tgfb1* and *Mcp1* mRNA (A), Tnf-α and Il-6 protein (B), mRNA expression of markers of HSC activation (*Acta2*, *Des*, *Gfap*) and KC activation (*Clec4f*, *Cd68*) (C), and *Mmp* (D) and *Timp* (E) mRNA in 12-mo-old male rat livers. Values are means ± SEMs; *n* = 10/group; 10/10 rats used. **^,^***C and R compared with CQ and RQ: **P < 0.01, ***P < 0.001. *^,^**C compared with R: *P < 0.05, **P < 0.01. ***R compared with RQ, *P* < 0.001. Statistical interactions: *Tgfb1*, *P* = 0.1; *Mcp1*, *P* = 0.3; Tnf-α, *P* = 0.5; Il-6, *P* = 0.002; *Acta2*, *P* = 0.5; *Des*, *P* = 0.3; *Gfap*, *P* = 0.5; *Clec4f*, *P* = 0.1; *Cd68*, *P* = 0.9; *Mmp2*, *P* = 0.9; *Mmp9*, *P* = 0.04; *Timp1*, *P* = 0.6; *Timp2, P* = 0.8. Data were analyzed by using 2-factor ANOVA and Duncan’s post hoc testing, where appropriate. *Acta2,* α-smooth muscle actin 2; C, control; *Cd68*, cluster of differentiation 68; *Clec4f*, C-type lectin-domain family 4; *Col1a1*, collagen type 1, α1; CoQ_10_, coenzyme Q; CQ, control CoQ_10_;* Des*, desmin; *Gfap*, glial fibrillary acidic protein;* Mcp1*, monocyte chemoattractant protein 1; *Mmp*, matrix metalloproteinase; R, recuperated; RQ, recuperated CoQ_10_;* Tgfb1*, transforming growth factor β1; *Timp*, tissue inhibitor of matrix metalloproteinase.

Recuperated and control offspring had similar expression of the profibrotic cytokine transforming growth factor β1 (*Tgfb1*), and a trend for greater expression of monocyte chemoattractant protein 1 (*Mcp1*; effect of maternal diet, *P* = 0.06) was observed in recuperated offspring (**Figure 2**A). CoQ_10_ supplementation reduced the concentrations of *Tgfb1* (effect of CoQ_10_ supplementation, *P* < 0.001) and *Mcp1* (effect of CoQ_10_ supplementation, *P* = 0.07) (Figure 2A). The cytokine Tnf-α was greater in recuperated offspring than in controls (effect of maternal diet, *P* < 0.05) and concentrations were reduced by CoQ_10_ supplementation (effect of CoQ_10_ supplementation, *P* < 0.05). Concentrations of Il-6 were greater (*P* < 0.05) in recuperated offspring than in controls and this effect was prevented by CoQ_10_ supplementation (*P* < 0.001; interaction between maternal diet and CoQ_10_ supplementation, *P* < 0.001) (Figure 2B). The gene expression of markers of hepatic stellate cell (HSC) and Kupffer cell (KC) activation [α-smooth muscle actin 2 (*Acta2*), desmin (*Des*), and C-type lectin-domain family 4 (*Clec4f*)] was greater in recuperated offspring than in controls (effects of maternal diet, *P* < 0.05 for all listed variables) (Figure 2C). CoQ_10_ supplementation reduced *Des* (*P* < 0.001), *Clec4f* (*P* < 0.05), and cluster of differientation 68 (*Cd68*) (*P* < 0.01; all were effects of CoQ_10_ supplementation) (Figure 2C). *Acta2* was unchanged by CoQ_10_ supplementation (Figure 2C). Glial fibrillary acidic protein (*Gfap*) mRNA expression was not significantly different between groups (Figure 2C). Gene expression of matrix metalloproteinase (*Mmp*) 9 (*Mmp9*) was greater (*P* < 0.01) in recuperated offspring than in controls, and CoQ_10_ supplementation reduced (*P* < 0.001) recuperated *Mmp9* mRNA to control levels (interaction between maternal diet and CoQ_10_ supplementation, *P* < 0.05) (Figure 2D). The gene expression of *Mmp2* remained unaltered between groups (Figure 2D). Tissue inhibitors of Mmps (*Timp*s) were also not significantly different between groups (Figure 2E).

### CoQ_10_ supplementation attenuates ROS induced by poor maternal nutrition

**FIGURE 3:**
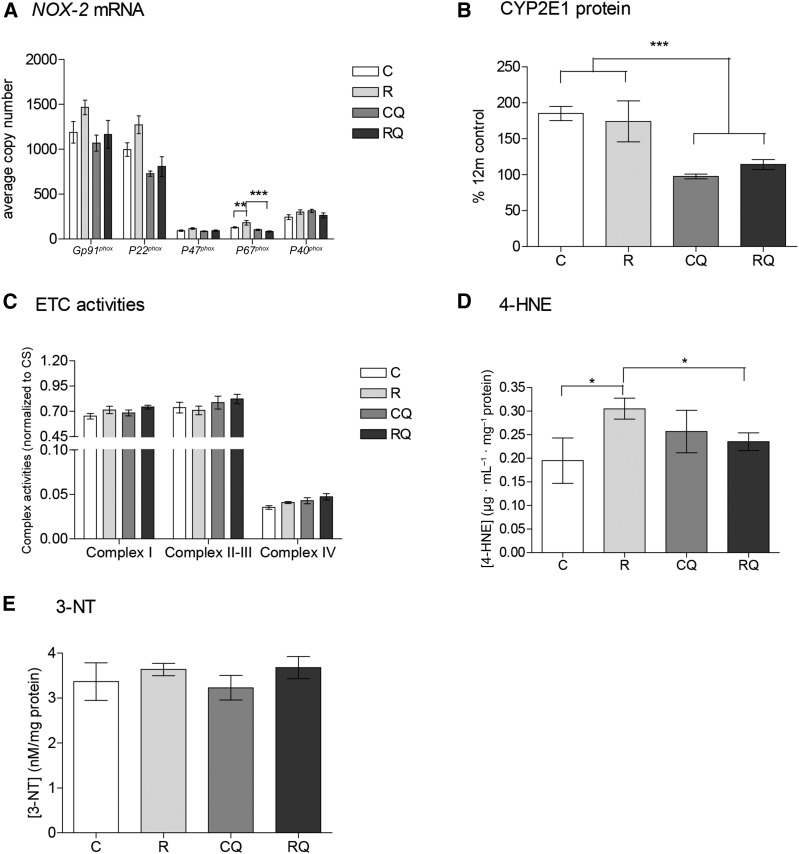
Effect of in utero protein restriction, accelerated postnatal growth, and CoQ_10_ supplementation on indexes of oxidative stress: components of the NOX-2 complex (A), CYP2E1 (B), ETC activities (C), 4-HNE (D), and 3-NT adducts (E) in 12-mo-old male rat livers. Values are means ± SEMs; *n* = 10/group; 10/10 rats used. D: *Comparison of C with R and comparison of R with RQ, *P* < 0.05. A and B: **Comparison of C with R, *P* < 0.01; ***Comparison of R with RQ and comparison of C and R with CQ and RQ, *P* < 0.001. Statistical interactions: NOX-2 (*Gp91^phox^*, *P* = 0.5; *P22^phox^*, *P* = 0.3; *P47^phox^*, *P* = 0.4; *P67^phox^*, *P* = 0.01; *P40^phox^*, *P* = 0.3), CYP2E1 (*P* = 0.5), ETC activities (complex I, *P* = 0.9; complexes II–III, *P* = 0.6; complex IV, *P* = 0.9), 4-HNE (*P* = 0.04), and 3-NT (*P* = 0.8). Data were analyzed by using 2-factor ANOVA and Duncan’s post hoc testing, where appropriate. C, control; CoQ_10_, coenzyme Q; CQ, control CoQ_10_; CS, citrate synthase; CYP2E1, cytochrome P450-2E1; ETC, electron transport chain; NOX-2, NAD(P)H oxidase 2; R, recuperated; RQ, recuperated CoQ_10_; 3-NT, 3-nitrotyrosine; 4-HNE, 4-hydroxynonenal.

Components of the NAD(P)H oxidase 2 (NOX-2) protein complex—*Gp91^phox^* (*P* < 0.05), *P22^phox^* (*P* < 0.05), and *P47^phox^* (*P* = 0.05)—were greater in recuperated offspring than in controls. *P67^phox^* was greater (*P* < 0.01) in recuperated offspring than in controls, and this effect was reduced (*P* < 0.001) by CoQ_10_ supplementation (interaction between maternal diet and CoQ_10_ supplementation, *P* < 0.01) (**Figure 3**A). Levels of *Gp91^phox^* (*P* = 0.08), *P22^phox^* (*P* < 0.001), and *P47^phox^* (*P* = 0.05) were reduced by CoQ_10_ supplementation (Figure 3A). CYP2E1 was not significantly different between control and recuperated offspring; however, CoQ_10_ supplementation reduced this concentration by 50% (effect of CoQ_10_ supplementation, *P* < 0.01) (Figure 3B). Complex I and complex IV electron transport chain activities were greater in recuperated offspring (effect of maternal diet, *P* = 0.05); however, complex II–III activity was unaffected. CoQ_10_ supplementation caused an increase in complex IV activity (effect of CoQ_10_ supplementation, *P* < 0.05) (Figure 3C). Mitochondrial DNA copy number was not significantly different between groups (control: 36 ± 4 copy numbers; recuperated: 36 ± 5 copy numbers) or by CoQ_10_ supplementation (control CoQ_10_: 41 ± 4 copy numbers; recuperated CoQ_10_: 35 ± 3 copy numbers). Concentrations of 4-hydroxynonenal (4-HNE) adducts were greater (*P* < 0.05) in recuperated offspring. There was a significant interaction between maternal diet and CoQ_10_ supplementation on 4-HNE concentrations (*P* < 0.05), reflecting the fact that CoQ_10_ supplementation reduced 4-HNE concentrations in recuperated offspring but had no effect in control offspring (Figure 3D). 3-nitrotyrosine concentrations were not significantly different between groups (Figure 3E).

### CoQ_10_ supplementation alters hepatic antioxidant defense capacity

**FIGURE 4:**
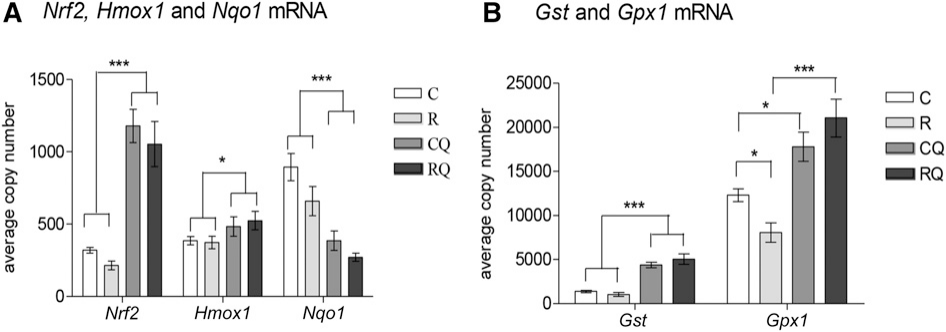
Effect of in utero protein restriction, accelerated postnatal growth, and CoQ_10_ supplementation on mRNA expression of molecules involved in the NRF antioxidant defense pathway in 12-mo-old male rat livers. (A) *Nrf2*, *Hmox1*, and *Nqo1* and (B) *Gst* and *Gpx1*. Values are means ± SEMs; *n* = 10/group; 10/10 rats used. *^,^***C and R compared with CQ and RQ: *P < 0.05, ***P < 0.001. *C compared with R and C compared with CQ, *P* < 0.05. ***R compared with RQ,* P* < 0.001. Statistical interactions: *Nrf2*, *P* = 0.9; *Hmox1*, *P* = 0.6; *Nqo1*, *P* = 0.4; *Gst*, *P* = 0.1; *Gpx1*, *P* = 0.01. Data were analyzed by using 2-factor ANOVA and Duncan’s post hoc testing, where appropriate. C, control; CoQ_10_, coenzyme Q; CQ, control CoQ_10_;* Gpx1*, glutathione peroxidase 1; *Gst*, glutathione synthetase; *Hmox1*, heme oxygenase 1; *Nqo1*, NAD(P)H dehydrogenase, quinone 1; NRF, nuclear erythroid 2-related factor; *Nrf2*, nuclear factor, erythroid 2–like 2; R, recuperated; RQ, recuperated CoQ_10_.

Nuclear factor erythroid 2–like 2 (*Nrf2)*, heme oxygenase 1 (*Hmox1*), and glutathione synthetase (*Gst*) expression were not significantly different between control and recuperated offspring (**Figure 4**A, B). Glutathione peroxidase 1 (*Gpx1*) was reduced in recuperated offspring compared with controls, and this effect was prevented by CoQ_10_ supplementation (interaction between maternal diet and CoQ_10_ supplementation, *P* < 0.05). NAD(P)H dehydrogenase, quinone 1 (*Nqo1*), was reduced in recuperated offspring (effect of maternal diet, *P* < 0.05) (Figure 4A, B). CoQ_10_ supplementation increased *Nrf2* (*P* < 0.001), *Hmox1* (*P* < 0.05), and *Gst* (*P* < 0.001) (Figure 4A, B); however *Nqo1* expression was reduced by CoQ_10_ supplementation (effect of CoQ_10_ supplementation, *P* < 0.001) (Figure 4A).

### CoQ_10_ supplementation alters expression of molecules involved in insulin and lipid metabolism

**FIGURE 5:**
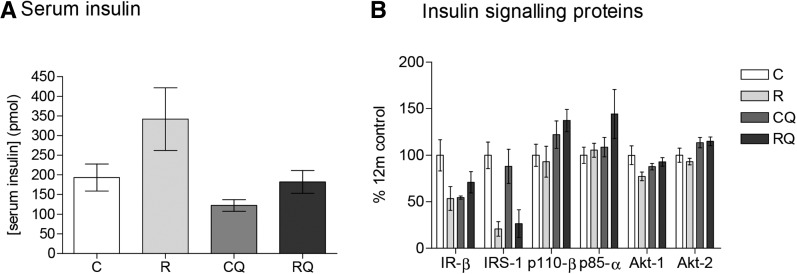
Effect of in utero protein restriction, accelerated postnatal growth, and CoQ_10_ supplementation on serum insulin (A) and insulin signaling protein expression (B) in 12-mo-old male rat livers. Values are means ± SEMs; *n* = 10/group; 10/10 rats used. Statistical interactions: insulin, *P* = 0.3; insulin signaling proteins (IR-β, *P* = 0.12; IRS-1, *P* = 0.5; p110β, *P* = 0.4; p85α, *P* = 0.3; Akt-1, *P* = 0.05; Akt-2, *P* = 0.5). Data were analyzed by using 2-factor ANOVA and Duncan’s post hoc testing, where appropriate. C, control; CoQ_10_, coenzyme Q; CQ, control CoQ_10_; IR-β, insulin receptor β IRS-1, insulin receptor substrate 1; p85α, phosphoinositide-3-kinase, p85-α p110-β, phosphoinositide-3-kinase, p110-β R, recuperated; RQ, recuperated CoQ_10_.

Greater serum insulin concentrations were observed in recuperated offspring than in controls (overall effect of maternal diet, *P* < 0.05) (**Figure 5**A). Concentrations were reduced by CoQ_10_ supplementation (effect of CoQ_10_ supplementation, *P* < 0.01) (Figure 5A). Protein expression of IR-β (*P* < 0.001), IRS-1 (*P* < 0.001), and Akt-1 (*P* < 0.05) was reduced in recuperated offspring compared with controls (all effects of maternal diet). Phosphoinositide-3-kinase-p110-β (p110-β), phosphoinositide-3-kinase-p85α (p85-α), and Akt-2 were not influenced by maternal diet (Figure 5B). CoQ_10_ supplementation increased p110-β (*P* < 0.05) and Akt-2 (*P* < 0.01) protein expression (Figure 5B). Fasting plasma glucose concentrations were not significantly different between groups (Table 3). Serum and hepatic triglyceride concentrations and serum cholesterol concentrations were not significantly different between control and recuperated offspring (Table 3). CoQ_10_ supplementation increased serum triglyceride and cholesterol concentrations (effects of CoQ_10_ supplementation, *P* < 0.05); however, hepatic triglyceride concentrations were unchanged (Table 3).

## DISCUSSION

Our findings that a maternal low-protein diet in utero followed by accelerated postnatal growth (recuperated) confers a higher risk of oxidative damage, proinflammatory changes, and liver fibrosis suggest that the early environment is an important determinant of an individual’s risk of developing complications of fatty liver disease. The potential for progression from NAFLD to nonalcoholic steatohepatitis and finally to hepatitis, fibrosis, and cirrhosis is well described. However, it has not previously been possible to identify patients who are at high risk of these changes. Our finding that recuperated rats developed more hepatic fibrosis than did controls indicates that the early environment plays a central role in the risk of liver disease in later life. Identifying the early environment as influential in the propensity to hepatic inflammation and fibrosis provides valuable new insight into predetermining individuals at increased risk from hepatic manifestations of the metabolic syndrome.

HSCs are the main source of extracellular matrix formation during hepatic fibrosis (26). The key step in inducing fibrosis during liver injury is the transformation of quiescent HSCs to activated HSCs, which differentiate into myofibroblasts (26). We found increased expression of *Acta2* and *Des* in recuperated offspring. *Acta2* is expressed in myofibroblasts of damaged liver (27) and hence is a good marker of HSC activation. In rats, HSC activation and proliferation correlate with a high expression of *Des* and are found in HSCs of acutely injured liver (27). KC infiltration and activation play a prominent role in HSC activation. Increased KC infiltration coincides with the activation of HSC markers such as *Acta2* (28). *Cle4f* (a unique KC receptor for glycoproteins and therefore a good marker of KC activation) was increased in livers of recuperated offspring.

Increased concentrations of proinflammatory cytokines are also crucial in initiating HSC activation. Hepatic protein expression of Tnf-α and Il-6 was greater in recuperated offspring, suggesting that inflammation plays a role in the HSC activation and consequent hepatic fibrosis observed in our model. *Tgfb1* mRNA levels were not changed in recuperated offspring; however, we cannot discount the possibility that *Tgfb1* is upregulated at the protein level. *Mcp1* (a chemokine that acts as a chemoattractant for HSCs) was also upregulated in recuperated livers. Mmps are also associated with hepatic fibrosis (29). *Mmp9* expression was upregulated in recuperated livers. Mmp9 is prominent in scar areas of active fibrosis, and treatment with a profibrotic agent can increase its expression, with peak expression coinciding with induction of inflammatory cytokines (29). *Mmp2* expression was unaltered between groups. Because *Mmp* expression is an early event in wound healing, the time window for *Mmp2* elevation may have been missed and that difference would be observed only in younger animals.

A further driving factor in HSC activation and fibrosis is increased ROS, which can be generated by Tnf-α, Il-6 (30), KCs (28), and the mitochondrial electron transport chain. We found increased ROS in the context of increased lipid peroxidation [which is known to increase in liver disease (31)] and greater expression of the NOX-2 components (*Gp91^phox^*, *P22^phox^*, *P47^phox^*, and *P67^phox^*), a major source of hepatic ROS production, which have been observed in hepatic fibrosis (32, 33). Complex I activity, a predominant generator of ROS (34), was greater in recuperated livers. Decreased antioxidant defense capacity was evidenced by a reduction in *Gpx1*, a peroxidase responsible for the conversion of H_2_O_2_ into H_2_O and O_2_. Increased concentrations of cellular H_2_O_2_, due to *Gpx1* depletion, could cause accumulation of the hydroxyl anion, a free radical that can directly increase lipid peroxidation.

Accumulation of hepatic triglycerides also plays a role in hepatic fibrosis (35); however, neither liver nor plasma triglycerides were altered in recuperated rats. This may be explained by the fact that recuperated offspring are fed a feed pellet diet and do not display an obesogenic phenotype. This in itself is interesting, because it shows that the observed deleterious liver phenotypes develop in a physiologic environment that had been influenced only by developmental programming per se, and not by obesity.

Insulin resistance and hyperinsulinemia are also major contributors to liver fibrosis (36) and are inherently linked to increased oxidative stress. Recuperated offspring had whole-body insulin resistance as indicated by hyperinsulinemia. The hyperinsulinemia was associated with hepatic insulin signaling protein dysregulation, as shown by the downregulation of IR-β, IRS-1, and Akt-1.

Importantly, we identified an effective means of arresting the pathologic progression of NAFLD, by postnatal supplementation with CoQ_10_. In recuperated offspring, CoQ_10_ supplementation reduced markers of HSC and KC activation, the accumulation of ROS, and the deposition of collagen around the hepatic vessels. This agrees with a study in which high-fat diet fed mice administered 1% CoQ_10_ supplementation led to reduced hepatic NOX expression (37). CoQ_10_ supplementation also increased activity of complex IV, in keeping with in vitro studies (38). CoQ_10_ supplementation decreased Tnf-α, Il-6, *Tgfb1*, and *Mcp1*, suggesting that CoQ_10_ also can reduce inflammatory changes in liver, which is consistent with studies in mouse liver (37) and human blood (39). Our data therefore recapitulate CoQ_10_’s function, both as a potent antioxidant (15) and as an anti-inflammatory agent.

CoQ_10_ supplementation also prevented hyperinsulinemia in recuperated rats; however, hepatic insulin signaling protein dysregulation was not normalized by CoQ_10_ supplementation. The whole-body insulin sensitivity may therefore be improved through other mechanisms such as improvements in muscle and/or adipose tissue insulin sensitivity. CoQ_10_ may exert antifibrotic effects through the activation of the Nrf2/antioxidant response element (Nrf2/ARE) pathway. Nrf2 is a transcription factor that responds to oxidative status and regulates transcription of genes involved in antioxidant defense. CoQ_10_ treatment in a model of hepatic fibrosis ameliorates liver damage via suppression of *Tgfb1* and upregulation of Nrf-ARE-associated genes (40). Although Nrf2 expression was not affected by maternal diet, CoQ_10_ supplementation increased Nrf2 by 4-fold. The antioxidant genes involved in the Nrf2/ARE pathway—*Hmox1*, *Gst*, and *Gpx1—*were increased by CoQ_10_ supplementation. This suggests that CoQ_10_ supplementation upregulates the Nrf2/ARE pathway via suppression of *Tgfb1* (40). These observations support a role for CoQ_10_ supplementation in increasing antioxidant defenses to a protective level in animals that have experienced detrimental catch-up growth (18, 19). Because Nqo1 activity is known to prevent 1 electron reduction in quinones, it is plausible that because hepatic CoQ_10_ concentrations are elevated by CoQ_10_ supplementation, *Nqo1* expression is not required and thus suppressed.

In conclusion, a suboptimal early-life environment combined with a mismatched postnatal milieu predisposes offspring to increased hepatic ROS, inflammation, and hyperinsulinemia, leading to hepatic fibrosis. This recapitulates changes seen in patients in whom benign NAFLD progresses to cirrhosis and ultimately liver failure. We suggest that the early environment life is crucial in identifying the subgroup of patients at highest risk of such progression; however, this should be tested in humans. A clinically relevant dose of CoQ_10_ reversed liver fibrosis via downregulation of ROS, inflammation, and hyperinsulinemia and upregulation of the Nrf2/ARE antioxidant pathway. Because fibrosis contributes to up to 45% of deaths in the industrial world (41), CoQ_10_ supplementation may be a cost-effective and safe way of reducing this global burden in at-risk individuals, before the development of an NAFLD phenotype.

## Footnotes

4 Abbreviations used: *Acta2*, α-smooth muscle actin 2; *Actb*, β-actin; ARE, antioxidant response element; *Cd68*, cluster of differentiation 68; *Clec4f*, C-type lectin-domain family 4; *Col1a1*, collagen type 1, α1; CoQ_9_, coenzyme Q isoform containing 9 isoprenoid units; CoQ_10_, coenzyme Q; CYP2E1, cytochrome P450-2E1; *Des*, desmin; EC, Enzyme Commission; *Gfap*, glial fibrillary acidic protein; *Gpx1*, glutathione peroxidase 1; *Gst*, glutathione synthetase; *Hmox1*, heme oxygenase 1; HSC, hepatic stellate cell; IR-β, insulin receptor β IRS-1, insulin receptor substrate 1; KC, Kupffer cell; *Mcp1*, monocyte chemoattractant protein 1; *Mmp*, matrix metalloproteinase; NAFLD, nonalcoholic fatty liver disease; NOX-2, NAD(P)H oxidase 2; *Nqo1*, NAD(P)H dehydrogenase, quinone 1; *Nrf2*, nuclear factor, erythroid 2–like 2; p85-α, phosphoinositide-3-kinase, p85-α p110-β, phosphoinositide-3-kinase, p110-β ROS, reactive oxygen species; *Tgfb1*, transforming growth factor β1; *Timp*, tissue inhibitor of matrix metalloproteinases; 4-HNE, 4-hydroxynonenal.
